# The *Plasmodium* palmitoyl-S-acyl-transferase DHHC2 is essential for ookinete morphogenesis and malaria transmission

**DOI:** 10.1038/srep16034

**Published:** 2015-11-03

**Authors:** Jorge M. Santos, Jessica Kehrer, Blandine Franke-Fayard, Friedrich Frischknecht, Chris J. Janse, Gunnar R. Mair

**Affiliations:** 1 Instituto de Medicina Molecular, Faculdade de Medicina da Universidade de Lisboa, Edifício Egas Moniz, Av. Prof. Egas Moniz, 1649-028 Lisbon, Portugal; 2 Parasitology, Department of Infectious Diseases, University of Heidelberg Medical School, Im Neuenheimer Feld 324, 69120 Heidelberg, Germany; 3 Leiden Malaria Research Group, Department of Parasitology, Leiden University Medical Center, Albinusdreef 2, 2333 ZA Leiden, The Netherlands

## Abstract

The post-translational addition of C-16 long chain fatty acids to protein cysteine residues is catalysed by palmitoyl-S-acyl-transferases (PAT) and affects the affinity of a modified protein for membranes and therefore its subcellular localisation. In apicomplexan parasites this reversible protein modification regulates numerous biological processes and specifically affects cell motility, and invasion of host cells by *Plasmodium falciparum* merozoites and *Toxoplasma gondii* tachyzoites. Using inhibitor studies we show here that palmitoylation is key to transformation of zygotes into ookinetes during initial mosquito infection with *P. berghei*. We identify DHHC2 as a unique PAT mediating ookinete formation and morphogenesis. Essential for life cycle progression in asexual blood stage parasites and thus refractory to gene deletion analyses, we used promoter swap (ps) methodology to maintain *dhhc2* expression in asexual blood stages but down regulate expression in sexual stage parasites and during post-fertilization development of the zygote. The *ps* mutant showed normal gamete formation, fertilisation and DNA replication to tetraploid cells, but was characterised by a complete block in post-fertilisation development and ookinete formation. Our report highlights the crucial nature of the DHHC2 palmitoyl-S-acyltransferase for transmission of the malaria parasite to the mosquito vector through its essential role for ookinete morphogenesis.

Malaria causing parasites have a complex life cycle alternating between a vertebrate host and a mosquito vector and are capable of invading diverse host cell types. Life cycle progression in the mosquito is initiated following a blood meal and the immediate differentiation of mature gametes that take part in sexual development; fertilisation of the female by the male gamete results in a diploid zygote that relies on maternally supplied gene products for the development of the next life cycle stage, the ookinete, within the next 24 hours^1^,^2^,^3^,^4^. Only the elongated and motile ookinete is capable of exiting the mosquito blood meal sack and traversing the midgut epithelium in order to settle as an extracellular oocyst where cell divisions result in the formation of thousands of sporozoites. Ookinete formation requires directional growth from a single point in the round, fertilised female gamete. This polarity is already visible in the round zygote and marked by proteins such as ISP1 and ISP3^5^; both proteins localise to the inner membrane complex (IMC) which is part of the larger ookinete pellicle that consists of the plasma membrane, the IMC and the subpellicular microtubules, also anchoring the gliding motility motor^6^. More than 40 proteins are associated with the pellicle^7^; many of those are translationally regulated prior to fertilization^1^ and post-translationally modified by the addition of lipids^8^. While prenylation and myristoylation are irreversible^9^, palmitoylation is reversible and thus can dynamically regulate a protein’s subcellular localization, gene expression and activity^8^,^10^,^11^,^12^,^13^,^14^. Palmitoylation results in the addition of a C-16 fatty acid to a cysteine residue within a given protein. Blocking palmitoylation in *P. falciparum* with 2-bromopalmitate (2-BMP)^15^,^16^ results in a complete failure to develop merozoites during the blood stage of the life cycle^8^. Preventing palmitoylation of proteins through targeted mutagenesis of cysteine residues within the modification target results in the mis-localization of proteins found in the IMC^17^. The palmitoylation reaction is catalysed by TM-spanning enzymes called palmitoyl-S-acyl-transferases (PAT). One family of PATs is characterised by the presence of a conserved DH(H/Y)C motif, and certain apicomplexan organisms express more than 10 individual S-acyltransferases^8^,^14^,^17^. They differ in localisation and timing of expression, and therefore are likely to modify distinct protein populations and biological functions. *Toxoplasma gondii* DHHC7 for example localises to rhoptry organelles and is necessary for invasion^14^,^18^, while tachyzoites treated with the PAT inhibitor 2-BMP^15^,^16^ show altered motility, invasion, replication and morphology^19^. Palmitoylation/de-palmitoylation can fine tune host cell invasion by *T. gondii*^20^.

The global extent of palmitoylation in asexual blood stages of *P. falciparum* comprises several hundred proteins; they include factors involved in gliding motility, invasion, adhesion, IMC function, signalling, protein transport and proteolytic activity^8^. Of 11 PATs known from rodent malaria parasites five have been detected in blood stage parasites of *P. berghei* using an HA-tagging approach^14^: they are DHHC3 (IMC), DHHC5 (ER), DHHC7 (rhoptry), DHHC8 (punctate_not_Golgi), and DHHC9 (IMC). Seven DHHC-PATs were found to be redundant for *P. berghei* blood stage development in a reverse genetic screen^14^: they are DHHC 3, 5, 6, 7, 9, 10 and 11; but none of these *P. berghei* DHHC mutants have been linked to a specific cell biological or developmental defect, and their functions remain thus elusive.

Six *P. falciparum* PATs were detected in proteomes of blood stages and salivary gland sporozoites^21^,^22^,^23^,^24^,^25^. Two (DHHC1 and DHHC4) have been detected in the ‘mixed gametocyte stage’ *P. berghei* proteome^26^, but not in zygotes and ookinetes^27^,^28^,^29^. The localisation patterns of some *P. berghei* PATs in blood stages suggest a role in motility, cell traversal or invasion^14^. The role of palmitoylation (if any) for ookinete and sporozoite development and its role for motility and invasion of host cells by these two life cycle stages remain however completely unknown. Capable of navigating both mosquito and mammalian host environments, these two life cycle forms are key to mosquito colonisation, invasion of its salivary glands and establishment of a liver infection. Drug inhibitor studies indicate here for the first time that protein palmitoylation plays an essential role in malaria parasite transmission, which we link by genetic studies to DHHC2; employing promoter swap methodology to modulate expression of DHHC2 during sexual development we demonstrate that DHHC2 is an essential factor for ookinete morphogenesis. DHHC2 is expressed in asexual blood stages (schizonts), during sexual development (in gametocytes, zygotes/ookinetes) and in sporozoites. This is the first report of a PAT knock-down mutant linked to a specific developmental defect in the malaria parasite life cycle.

## Results and Discussion

### Palmitoylation is essential for zygote to ookinete transformation

**Figure 1:**
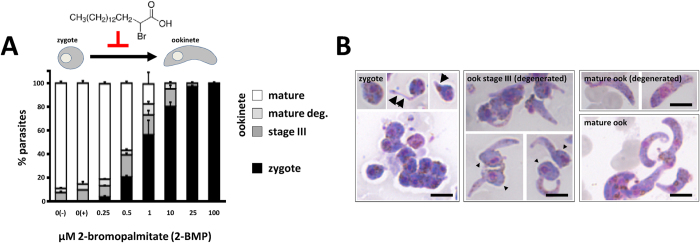
Inhibition of protein palmitoylation blocks zygote development. (**A**) The effect of 2-bromopalmitate (2-BMP) on ookinete formation is dose-dependent. Control cultures were set up in the presence (+) and absence (−) of DMSO. Data represent mean ± SEM. (**B**) Representative images of the Giemsa-stained developmental stages observed in cultures from (**A**). Stage I/II zygotes often show small protrusions (arrows), while degenerated stage III ookinetes are identified by long protrusions and a posterior bulb containing the nucleus (arrowheads). Scale bars = 5 μm.

Following transmission of *Plasmodium* parasites to the mosquito vector during a mosquito blood meal, gametes mate to form a round zygote which subsequently develops into a banana-shaped, motile ookinete. To investigate whether palmitoylation is needed for this transformation process, we evaluated the effect of the S-acyl-transferase inhibitor 2-BMP on *P. berghei* ookinete development *in vitro* using conditions established for 2-BMP inhibition of blood stages of *P. falciparum in vitro*^8^. 2-BMP was added in a 0–100 μM range to standard *in vitro* ookinete cultures^30^ one hour after gametocyte activation—this corresponds to the time point when gamete formation and fertilisation are completed; the formation of mature ookinetes was quantified 18 hours later. In control cultures without drug (vehicle only) a mean of 77% (range 68–89%, *n* = 3) of female gametocytes were fertilised and had developed into mature ookinetes with wild-type morphology. Addition of 2-BMP produced a clear dose-dependent effect on the transformation of zygotes into mature ookinetes (Fig. 1A). Zygote to mature ookinete development was completely blocked at concentrations of 100, 25 and 10 μM resulting in the absence of mature ookinetes. In these cultures only clusters of round zygotes or zygotes with small, thin protrusions were present indicating initiated, but failed morphogenesis (Fig. 1B); at 10 μM 2-BMP approximately 20% of the parasites developed into cells with prolonged protrusions (retort stage; stage III) but none produced fully mature, banana-shaped ookinetes; at 1 μM 2-BMP less than 20% of parasites were able to form morphologically mature ookinetes whereas the remaining parasites were arrested at the zygote/retort stages (Fig. 1B); as little as 0.25 μM 2-BMP was effective in interfering with ookinete formation indicating a comprehensive role for protein palmitoylation in zygote to ookinete transformation.

### *P. berghei* DHHC2 expression and evidence for distinct mRNA pools

**Figure 2:**
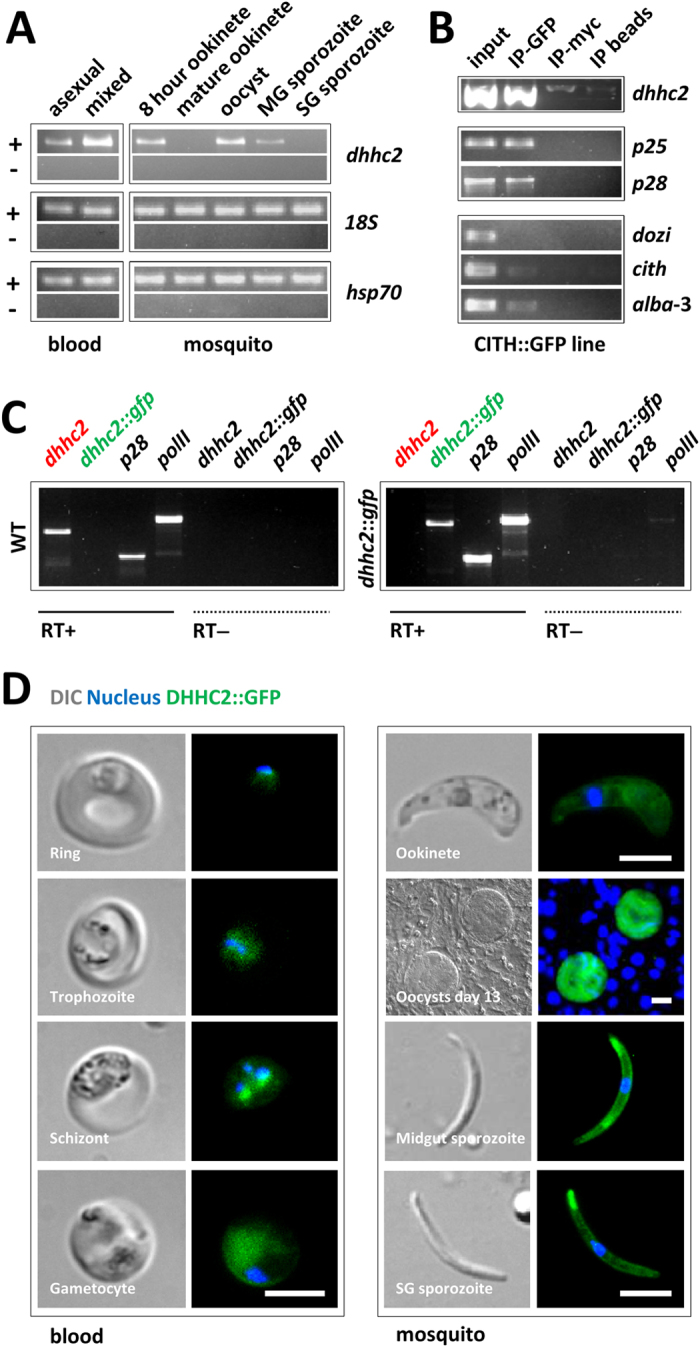
*dhhc2* is expressed throughout the parasite life cycle and associates with the translational repressor CITH in gametocytes. (**A**) RT-PCR analysis of *dhhc2* across the life cycle reveals widespread transcription. asexual: asexual blood stages; mixed: asexuals & gametocytes; MG: midgut; SG: salivary gland. *18S ribosomal RNA* and *hsp70* serve as loading controls. (**B**) RT-PCR analysis of CITH::GFP gametocyte immunoprecipitation (IP) eluates shows that *dhhc2* is bound by CITH like *p25* and *p28*. Translated mRNAs (*dozi*, *cith* and *alba*-3) are not enriched in the IP-GFP fraction. Input: total gametocyte mRNA; IP-GFP: IP with anti-GFP antibody; IP-myc: IP with anti-c-myc antibody; IP beads: no antibody used for IP. (**C**) RT-PCR analyses confirm exclusive transcription from the mutant GFP-tagged allele in mixed blood stages. *p28* and RNA polymerase II serve as control genes. (**D**) Expression of DHHC2::GFP in live *dhhc2::gfp* parasites. Scale bar = 5 μm (blood stages, ookinetes and sporozoites) or 20 μm (oocysts).

Like for *P. falciparum* merozoite formation^8^ protein palmitoylation is likely required for ookinete formation. Which of the of PAT(s) is (are) involved, is unknown. Ookinete formation depends on maternally provided (translationally repressed) mRNAs^1^,^2^,^3^,^4^ and three PATs are under putative translational control in the female gametocyte: *dhhc2*, *dhhc3* and *dhhc10*^3^. Only *dhhc2* (PBANKA_010830) is also transcribed in asexual stage parasites (where it perhaps mediates merozoite formation) and is present well into ookinete formation at least 8 hours after fertilisation (Fig. 2A). We analysed *dhhc2* (PBANKA_010830) transcriptional patterns by semi-quantitative RT-PCR using cDNA prepared from blood and mosquito stage parasites; compared to the control transcripts of *hsp70* and *18S ribosomal RNA,* we found *dhhc2* to be highly transcribed in asexual blood stage parasites and in gametocytes, while mRNA levels were absent in mature ookinetes; in oocysts/midgut sporozoites the gene was transcribed anew but absent in mature salivary gland sporozoites (Fig. 2A). An RNAseq analysis of different *P. berghei* life cycle stages^31^ also found high transcript levels in schizonts and gametocytes compared to ring forms and trophozoites (Figure S1). Together, these data suggested a role for DHHC2 across several parasite life cycle stages in the mammalian and mosquito host including the gametocyte, zygote and developing ookinete.

DHHC2 is highly conserved across the genus *Plasmodium* (Figure S2) and related to PATs from apicomplexans such as *T. gondii* (Figure S3) with its four conservatively positioned transmembrane domains and central DH(H/Y)C-motif.

The high mRNA levels in gametocytes along with a concomitant reliance of the transcript for stability on translational repression^3^ suggested that *dhhc2* could be subjected to translational silencing in gametocytes and maternally provided to the developing ookinete. Like for *p25* and *p28*, two well-known translationally repressed transcripts^1^,^2^,^3^, an RNA-immunoprecipitation (RIP) experiment confirmed the association of *dhhc2* with the translational repressor CITH in gametocytes (Fig. 2B).

To assess protein expression, we targeted *dhhc2* for *in situ* GFP-tagging with a construct that integrates into the genome by single cross-over homologous recombination. We were able to select a *dhhc2::gfp* mutant (Figure S4) that transcribes only the tagged gene (Fig. 2C). *dhhc2::gfp* mutants produced wild type numbers of gametocytes (17.6 ± 1.5%; *n* = 3) and ookinetes (76.7 ± 11.0%; *n* = 3), and completed mosquito passage to a subsequent rodent host with a prepatent period of 4 days comparable to wild type (*n* = 1). Expression of the DHHC2::GFP protein in blood and mosquito stage parasites was determined by live fluorescence microscopy (Fig. 2D). GFP-positive (GFP+) signal was strongest in schizonts and gametocytes; in ookinetes the staining appeared weaker than in oocysts and sporozoites.

Translation of *dhhc2::gfp* in gametocytes indicated that the *dhhc2* 5′ UTR was not able to silence translation and that it may instead require the 3′ UTR akin to the ookinete surface protein *p28*^32^. The size of the 3′ UTR of *dhhc2* was determined by 3′ rapid amplification of cDNA ends (RACE) and found to extend over 495 nucleotides downstream of the UAG stop codon excluding the poly(A) tail (Figure S5A). However, a second GFP-tagged mutant (*dhhc2::gfp::3*′*utr*), this time including also the entire endogenous *dhhc2* 3′ UTR (plus an additional 43 base pairs) in the plasmid construct (Figure S5B–D), showed the same protein expression profile and did not abrogate protein expression in gametocytes (Figure S6). Considering the RIP data that showed a clear association of this transcript with the translational repressor CITH, protein expression in gametocytes was unexpected. Genes like *p25* and *p28*, and novel members of this large RNA regulon that were experimentally tested recently^1^, are not translated in *P. berghei* gametocytes. Our results here indicate that protein translation of transcripts associated with DOZI and CITH could be more intricately regulated than previously thought, perhaps allowing translation of mRNAs early during gametocyte formation when they are not yet bound by repressors, while silencing them later in mature gametocytes. In the *dhhc2::gfp::3*′*utr* mutant we observed stronger expression in ookinetes compared to *dhhc2::gfp* ookinetes with a punctate appearance. In oocysts the staining was diffuse, while it appeared peripheral in midgut and salivary gland sporozoites as in *dhhc2::gfp* parasites.

### *dhhc2* is refractory to gene deletion

In order to identify a biological function for DHHC2 we next attempted the generation of null mutants. Using standard methods of *P. berghei* genetic modification^33^ we targeted *dhhc2* for deletion by double cross-over homologous recombination of a plasmid containing a *T. gondii* dihydrofolate reductase/thymidylate synthase (*tgdhfr*/*ts*) selectable marker. Three independent transfection experiments for *dhhc2* were unsuccessful, pointing towards a vital role in invasion, growth or multiplication of asexual blood stages. This was not surprising given the expression profiles identified for both mRNA and protein in *P. berghei* (Fig. 2A,D and Figure S6) as well as in *P. falciparum*^17^ with very clear evidence for blood stage protein expression. Whether *P. berghei* DHHC2 is essential was not determined in an earlier study, but found the *T. gondii* DHHC2 homolog to be vital^14^.

### Promoter swap supports asexual stage growth and gametocyte formation, but results in ookinete formation failure

**Figure 3:**
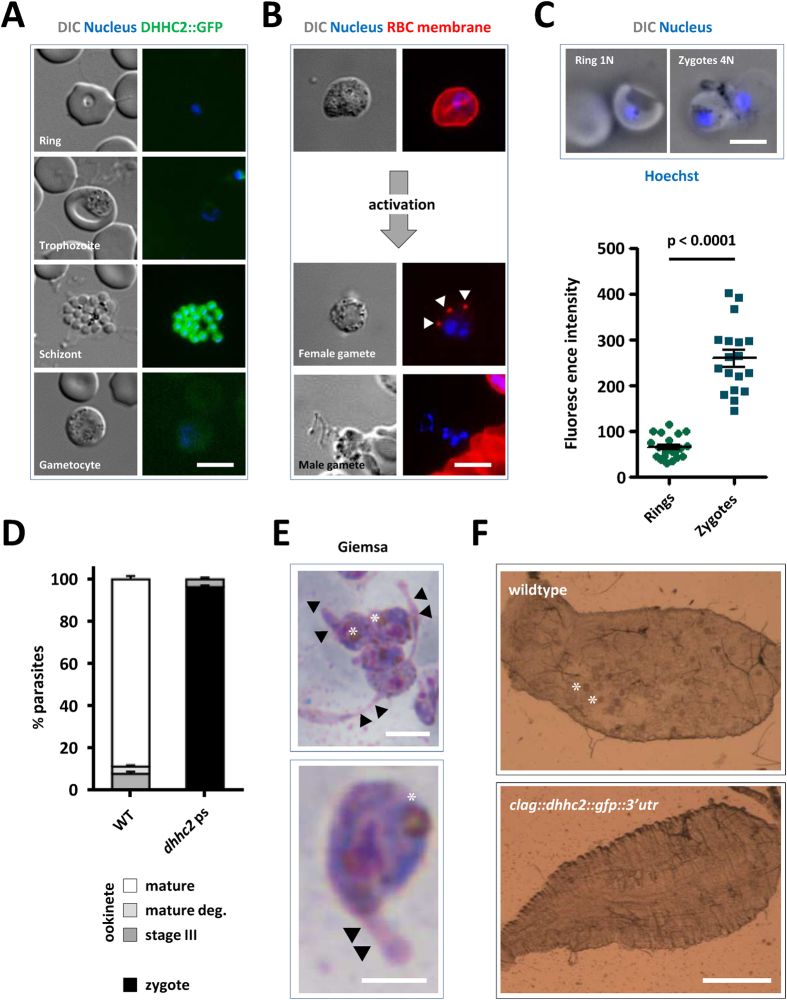
*clag::dhhc2::gfp::3*′*utr* parasites express DHHC2 in asexuals but not gametocytes, and fail to form ookinetes nor establish mosquito infections. (**A**) Expression of DHHC2::GFP in live parasites. Scale bar = 5 μm. (**B**) Male and female gametes egress normally in the *dhhc2* promoter swap line. RBC membrane highlighted by the marker TER-119 reveals normal female egress; note the remnants of the RBC membrane (arrowheads) adjacent to the free gamete. In males, DNA replication and formation of individual nuclei is evident. Scale bar = 5 μm. (**C**) Live imaging of Hoechst-stained ring stage and zygotes of the *dhhc2* promoter swap line (scale bar = 5 μm), and quantification of DNA content. Nuclear DNA content of zygotes of *clag::dhhc2::gfp::3*′*utr* parasites of asexual blood stage rings (haploid DNA content, 1N) and zygotes (4N) was determined by Hoechst-fluorescence intensity measurements. The mean fluorescence intensity of ring-form nuclei (1N) is 61 (*n* = 20) and of zygotes (4N) is 259 (*n* = 18). Mean ± SEM values and p-value for Student’s *t*-test are shown. (**D**) Quantification of ookinete formation defects in *clag::dhhc2::gfp::3*′*utr* mutants. Data represent mean ± SEM. (**E**) Giemsa staining of overnight ookinete cultures reveals 2-BMP-like morphological defects in mutant zygotes displaying thin protrusions (arrowheads). Asterisk labels the crystalloid. Scale bar = 5 μm. (**F**) Representative images of *A. stephensi* midguts 12 days after a blood meal on wildtype and *clag::dhhc2::gfp::3*′*utr* mutants. Asterisks indicate two individual of the many oocysts present in a wildtype-infected mosquito; none are visible in the mutant-infected. Scale bar = 0.5 mm.

Unable to generate a gene deletion mutant we next used promoter swap methodology^4^,^34^ to generate a mutant that expresses the GFP-tagged version of DHHC2 under the asexual stage-specific promoter of the cytoadherence linked asexual (protein) gene (*clag*) PBANKA_140060. The *clag* gene, like *dhhc2* is strongly transcribed in schizonts, but silent in gametocytes and ookinetes (Figure S7)^4^,^31^. Mutants were generated by introducing a plasmid construct by double cross-over homologous recombination into the *dhhc2::gfp::3*′*utr* line (Figure S8) to allow altered protein translation intensities in the different life cycle stages to be monitored by microscopy. Selection with WR99210 yielded the following mutant: *clag::dhhc2::gfp::3*′*utr*. Live microscopy identified GFP+ asexual cells with a strong signal in schizonts and individual merozoites; no signal was evident from other asexual stage parasites or sexual stage parasites as expected from using the *clag* promoter (Fig. 3A; compare with Fig. 2D and Figure S6). The asexual multiplication rate per 24 hours of blood stage parasites in mice infected with a single parasite of this clone was normal at 10 ± 0, and thus the promoter swap fully supported asexual development of the parasite.

The mutant strain generated male and female gametocytes that emerged normally following transfer into ookinete medium. Gamete formation appeared normal: microgametocytes differentiated into exflagellating male gametes; female macrogamete egress was evidenced by the remnants of the lysed red blood cell membrane stained for the RBC marker Ter119 (Fig. 3B). Nonetheless, *clag::dhhc2::gfp::3*′*utr* parasites failed to generate ookinetes in three independent experiments suggesting a fertilization or developmental defect. Overnight ookinete cultures stained with Hoechst however showed signs of fertilisation. Image analyses of ploidy confirmed that fertilisation had not been affected with parasite DNA content of these forms being approximately 4N; the zygotes have thus completed meiotic DNA replication to tetraploidy (Fig. 3C)^2^,^30^. However, none of these zygotes developed into mature ookinetes (Fig. 3D) and presented morphogenetic defects (Fig. 3E) resembling those seen following treatment with 2-BMP (compare with Fig. 1B). Two independent infections of mosquitoes with *clag::dhhc2::gfp::3*′*utr* parasites did not result in the establishment of oocysts (*n* = 20) (Fig. 3F), while control infections with wild type parasites resulted in normal oocyst development [213 ± 155 (mean ± s.d.); *n* = 20]. We confirmed the developmental defect during ookinete development in an independent mutant. This mutant was generated by transfection of the wild type *P. berghei* reference line 676m1cl1^35^ using the same promoter swap plasmid (Figure S9). Together the data clearly show that following fertilization DHHC2 has a key function in zygote development and ookinete differentiation.

## Conclusions

Our studies provide conclusive genetic evidence for the essential nature of a specific palmitoyl-S-acyltransferase (DHHC2) for malaria parasite life cycle progression in the mammalian host and transmission to the mosquito vector. Global inhibition of PATs with 2-BMP and a gene deletion screen had already established palmitoylation and certain S-acyltransferases to be required for asexual blood stage development^8^,^14^, although the specific role of any given enzyme had not been reported; their essential functions were deduced from the observation that gene deletion mutants were not viable^14^. Here our data clearly show the explicit involvement of the S-acyltransferase DHHC2 in life cycle progression of the rodent malaria parasite in the mosquito vector. Our results highlight a very distinct biological process—ookinete morphogenesis—to require the palmitoyl-S-acyl-transferase DHHC2. The inhibition of palmitoylation during blood stage and ookinete development (through the use of 2-BMP or the deletion of dhhc2) has similar consequences—a rather late morphological blockade. Treatment of *P. falciparum in vitro* cultures with 2-BMP results in schizont developmental defects caused by the disruption of intracellular membranes that individualise future merozoites, and thus impedes subsequent red blood cell invasion by newly released parasites through the destabilisation of gliding motor components like GAP45 and MTIP^8^. The shape of *Plasmodium* merozoites and ookinetes but also sporozoites depends on the ordered arrangement of the subpellicular network and the inner membrane complex (IMC) beneath the plasma membrane. At least 23 proteins are known to localise to the subpellicular network or are associated and inserted into the IMC; fourteen of those were found to be palmitoylated in *P. falciparum* asexual blood stage parasites^8^ and could be targets of this post-translational modification also in the developing zygote/ookinete. Our report serves as a clear starting point for further experiments to identify protein targets that require DHHC2-mediated palmitoylation for ookinete development. The GFP tagging experiments and the inability to generate a gene deletion mutant supporting asexual parasite growth likely indicate that the protein plays a similar developmental role during intraerythrocytic merozoite and sporozoite formation in oocysts of the mosquito. Affecting transmission between the mammalian host and mosquito vector, the development of parasite-specific PAT inhibitors could be of interest for novel malaria intervention strategies.

## Methods

### Ethics statement

All animal experiments in this study were carried out in accordance with the European Guideline 86/609/EEC and follow the FELASA (Federation of European Laboratory Animal Science Associations) guidelines and recommendations concerning laboratory animal welfare. Animal experiments performed in Leiden University Medical Center (LUMC, Leiden, The Netherlands) were approved by the Animal Experiments Committee of the Leiden University Medical Center (DEC 10099; 12042; 12120). Animal experiments performed at the University of Heidelberg Medical School (Heidelberg, Germany) were approved by the German Authorities (Regierungspräsidium Karlsruhe, Germany), § 8 Abs. 1 Tierschutzgesetz (TierSchG) (Aktenzeichen 35-9185.81/G-3/11). Animal experiments performed at Instituto de Medicina Molecular (IMM, Lisbon, Portugal) were approved by the IMM Animal Ethics Committee (under authorisation AEC_2010_018_GM_Rdt_General_IMM), the Portuguese authorities (Direção Geral de Alimentação e Veterinária) and were done in compliance with the Portuguese Law (Portaria 1005/92).

### Experimental animals

Female Balb/c ByJ and OF-1 (6–8 weeks bred at Charles River, France) mice were used and kept under standard conditions. Normal Chow and water were provided *ad libitum*.

### Reference *P. berghei* ANKA lines

The following reference *P. berghei* ANKA parasite lines were used; details can be found in the RMgm database (www.pberghei.eu). Line 909cl1 (CITH::GFP; RMgm-358)^2^ expressing a C-terminally GFP-tagged version of the translational repressor CITH (PBANKA_130130); line HPE, a non-gametocyte producer clone^36^; line 820cl1m1cl1 (Fluo-frmg; RMgm-164)^2^ expressing RFP under the control of a female gametocyte specific promoter and GFP under the control of a male gametocyte specific promoter; line 259cl1 (PbGFPcon; RMgm-5) expressing GFP under the control of the constitutive *eef1a* promoter; line 676m1cl1 (PbGFP-LUCcon; www.pberghei.eu, RMgm-29) expressing the fusion protein GFP-Luciferase under the control of the constitutive *eef1a* promoter; and line cl15cy1, which is the reference parent line of *P. berghei* ANKA^35^. Lines Fluo-frmg and PbGFP-LUCcon contain the transgene integrated into the silent *230p* gene *locus* (PBANKA_030600) and do not contain a drug-selectable marker.

### Oligonucleotides

Primers used in this study are listed in Table S1.

### RNA-immunoprecipitation (RIP) and Reverse Transcriptase-PCR

Immunoprecipitation (IP) of CITH::GFP parasite lysates, and subsequent RNA extraction and RT-PCR were performed as described^2^. To investigate the transcription patterns of the different *dhhc* genes by RT-PCR, RNA from different life cycle stages were obtained using TRIzol^®^ Reagent (Ambion^®^, #15596). Reverse transcription was performed with random primers and oligo-d(T) using SuperScript^®^ II Reverse Transcriptase (Invitrogen™, #18064). RNA sample origins were as follows: asexual blood stages from line HPE; mixed blood stages (containing asexual parasites and gametocytes) and oocysts d12 p.i. from line Fluo-frmg; *in vitro* cultured ookinetes at 16 hours after gametocyte activation from line cl15cy1; and midgut and salivary gland sporozoites day 20 p.i. from line PbGFPcon. Primers used in RT-PCRs are shown in Table S1.

### 3′ UTR determination of *dhhc2* by Rapid amplification of cDNA ends (RACE)

To identify the length of the 3′ UTR of *dhhc2* we employed 3′ RACE. Briefly, cDNA was prepared as above from mixed blood stage infections, followed by first-round amplification^37^ with Taq polymerase and the forward primer g3315, which spans an exon-exon junction; this was followed by nested PCR with forward primers 1301 and oligo d(T) primer 0357, cloning of the product into pGEM T-Easy and sequencing with T7, SP6 and two gene-specific primers (3317 and 3318).

### Generation of transgenic lines expressing GFP-tagged DHHC2

*In situ* C-terminal GFP-tagging of *dhhc2* was performed by single cross-over homologous recombination into the *dhhc2* locus. See Table S2 and Figures S4 and S5 for the name and details of the plasmid constructs pLIS0086 and pLIS0202. Both constructs contain the *tgdhfr*/*ts* selectable marker. Primers 0646 and 0644 were used to amplify the targeting region of *dhhc2*, corresponding to the 3′ end of the open reading frame (ORF) excluding the stop codon, as well as the 3′ UTR (primers 0741 and 0742) of *dhhc2* are listed in Table S1. Linearised plasmids (FspAI or BsmI) were transfected into cl15cy1 parasites using standard methods. Transfection, selection and cloning of mutant parasite lines was performed as described^33^, resulting in the following clonal lines: 2185cl1m1 (*dhhc2*::*gfp*) and 202cl1m1 (*dhhc2*::*gfp*:*:3*′*utr*). Correct integration of the constructs was confirmed by diagnostic genotyping PCR (Figures S4 and S5).

### Generation attempt for a *dhhc2* gene deletion mutant

To disrupt *dhhc2* (PBANKA_010830) we constructed a replacement construct which contains the pyrimethamine resistant *tgdhfr/ts* as a selectable marker cassette flanked by target sequences for homologous recombination; they were PCR-amplified from *P. berghei* WT genomic DNA using primers 0739 and 0740, and 0741 and 0742 specific for the 5′ or 3′ flanking regions of the *dhhc2* ORF, respectively. See Tables S1 and S2 for details of primers and the plasmid construct. The final DNA construct pLIS0065 used for transfection was obtained after digestion of the replacement construct with Asp718I and NotI. Transfection and selection of mutant parasite lines were performed as described^33^.

### Generation of promoter swap transgenic line expressing GFP-tagged DHHC2

pLIS0209 (Figure S8) contains the entire *dhhc2* ORF (amplified with primers 3010 and 0644) and promoter sequence from the cytoadherence linked asexual (protein) gene (*clag*) PBANKA_140060 (amplified with primers 3024 and 3025). The human *dhfr* selection marker is flanked by a second upstream targeting region (amplified with primers 0739 and 0740) to allow for double cross-over homologous recombination following digestion with KpnI and BglI. Transfection of line 202cl1m1 with pLIS0209 was followed by WR99210 selection on four consecutive days; a final clonal line (209cl2) was established by limiting dilution^33^.

### Generation of promoter swap transgenic line expressing non-tagged DHHC2

Construct pLIS0209 (see Figure S8 and Table S2) was transfected into 676m1cl1 parasites using standard methods after digestion with *Kpn*I and *Bam*HI. Transfection was followed by WR99210 selection on four consecutive days, resulting in the parasite line 2593 (*clag*::*dhhc2*). Correct integration of the construct was confirmed by Southern analysis of FIGE-separated chromosomes with a probe recognising the human *dhfr* present on the transfection plasmid (Figure S9).

### Ookinete formation assays and palmitoyl-S-acyl-transferase inhibition during ookinete development

*In vitro* fertilisation and ookinete formation assays were performed following published methods using gametocyte-enriched blood collected from mice treated with phenylhydrazine/NaCl^38^. Briefly, infected blood containing gametocytes was mixed in standard ookinete culture medium in 24-well plates and cultures were incubated for 18–24 h at 21–22 °C. To inhibit palmitoylation during ookinete development, 2-BMP (Sigma-Aldrich^®^, #21604) was added 1 hour after adding *dhhc2*::*gfp* infected blood containing gametocytes to standard ookinete cultures as described above. 2-BMP was added at final concentrations of 0, 0.25, 0.5, 1, 10, 25 and 100 μM. Since 2-BMP is prepared in DMSO, 0 μM 2-BMP control cultures were set up in the presence (+) and absence (−) of DMSO. Eighteen hours after drug addition the different developmental stages (zygotes, developing ookinetes and mature ookinetes) were counted in Giemsa-stained slides according to the classification in^30^. Percentages from 2 independent experiments are presented for 0 (+), 0.25, 0.5, 25 and 100 μM 2-BMP, while data for 0 (−), 1 and 10 μM 2-BMP are from 4 independent experiments.

### Live imaging of blood stages, ookinetes, oocysts and sporozoites

Live imaging of transgenic parasites expressing GFP-tagged DHHC2 was done by collecting tail blood samples from infected mice, mosquito blood meals at 16 hours p.i., as well as dissected mosquito midguts, midgut sporozoites and salivary gland sporozoites and staining with 1 μg/mL of Hoechst 33342/PBS. Red blood cell membranes were stained with anti-mouse TER-119 antibody conjugated with Alexa-488 (BioLegend; 1/500). Images were taken with a Leica DM5000B or Zeiss Axiovert 200M fluorescence microscope and processed using ImageJ 1.47n software (imagej.nih.gov/ij).

### Oocyst production

Oocyst production was analysed by performing standard mosquito infections. Naïve female Balb/c ByJ mice were infected intraperitoneally (IP) with 10^6^ infected red blood cells (iRBCs). On days 4–5 p.i., these mice were anesthetised and *Anopheles stephensi* female mosquitoes allowed to feed for 30 min. Twenty-four hours after feeding, mosquitoes were anaesthetised by cold shock and unfed mosquitoes were removed. Oocyst numbers were counted at day 12 after mosquito infection.

### DNA content quantification of nuclei of rings and zygotes

For nuclear DNA content quantification of mutant zygotes, live parasites from overnight *in vitro* ookinete cultures were stained with the DNA-specific dye Hoechst 33342 (Sigma, NL; 2 μmol/L) for 20 minutes. Pictures of zygotes (cells with protrusions) were taken with a LeicaDM/RB microscope (1000x magnification, oil RC immersion objective). Asexual blood stage ring forms (haploid DNA content; 1N) were used as control. The integrated density (IntDen) of Hoechst-stained nuclei in the ring forms and zygotes were measured using Image J 1.47n software (imagej.nih.gov/ij). Statistical analyses of DNA content in rings versus zygotes was performed using Student´s *t*-test as part of Prism software package 5 (GraphPad Software).

### Multiple sequence alignments

Protein sequences in Figures S2 and S3 were retrieved from PlasmoDB (plasmodb.org) and ToxoDB (toxodb.org). Clustal W alignments were performed at the EMBnet server (embnet.vital-it.ch/software/ClustalW.html) and shaded according to protein similarity levels with BOXSHADE 3.21 (www.ch.embnet.org/software/BOX_form.html).

## Additional Information

**How to cite this article**: Santos, J. M. *et al.* The *Plasmodium* palmitoyl-S-acyl-transferase DHHC2 is essential for ookinete morphogenesis and malaria transmission. *Sci. Rep.* **5**, 16034; doi: 10.1038/srep16034 (2015).

## Supplementary Material

Supplementary Information
