# Supplementary material for: The Plasmodium palmitoyl-S-acyl-transferase DHHC2 is essential for ookinete morphogenesis and malaria transmission
Source: Sci Rep. 2015 Nov 3;5:16034. doi: 10.1038/srep16034 (PMC4630622; doi:10.1038/srep16034)
Supplement: Supplementary Information [file srep16034-s1.pdf]

The *Plasmodium* palmitoyl-S-acyl-transferase DHHC2 is essential for ookinete morphogenesis and malaria transmission

Jorge M. Santos, Jessica Kehrer, Blandine Franke-Fayard, Friedrich Frischknecht, Chris J. Janse, Gunnar R. Mair

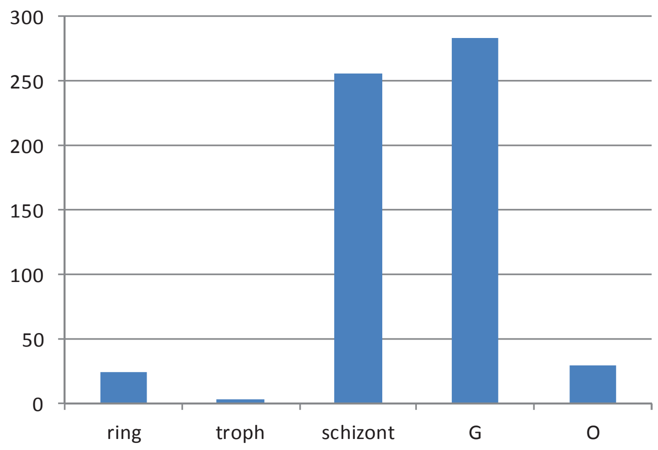

Supplementary Figure S1. PBANKA_010830 transcript levels as reads per kilobase of exon model per million mapped reads (RPKM). Ring 4-hour ring stage; troph 16-hour trophozoite; schizont 22-hour schizont; G gametocytes; O 24-hour ookinete; Otto et al. 2014

```
PBANKA_010830      1 MTHKYMQISHGQPEKKRGGSIFIFIVFFILSFIYIGYTGIVLRSWFIPYR
PCHAS_010890       1 MTHKYMQISHGQPEKKRGGSIFIFIVFFILSFIYIGYTGIVLRSWFIPYR
PY17X_0109900      1 MTHKYMQISHGQPEKKKGGSIFIFIVFFILSFIYIGYTGIVLRSWFIPYR
PKNH_1140400       1 -----MQPLQGQTEKKKRASIFIFIVFFVQAFIYVGYTGIVLRSWFIPYR
PVX_113640         1 MRPKYMQPAQGQTEKKKGGSIFIFIVFFILTFIYIGYTGIVLRSWFIPYR
PF3D7_0609800      1 MRPKYVQASQGQKEKKRGGSLFIFIVFFVLSFIYIGYTGIVLRSWFIPYR
PRCDC_0608500      1 MRPKYVQASQGQKEKKRGGSLFIFIVFFVLSFIYIGYTGIVLRSWFIPYR

PBANKA_010830     51 SGSLTIAIVFHIFFCLFLLSFIKCASTDPGKVPRNWGFYVGDDVKRRRYC
PCHAS_010890      51 SGSLTIAIAFHIFFFLFLLSFIKCASTDPGKVPRNWGFYVGDDVKRRRYC
PY17X_0109900     51 SGSLTIAIAFHIFFCLFLLSFIKCASTDPGKVPRNWGFYVGDDVKRRRYC
PKNH_1140400      46 SGSLTIAALFHVFFFLFLLSFIKCASTDPGKVPRNWGFYIGDDVKRRRYC
PVX_113640        51 SGSFTIAALFHVFFFLFLLSFIKCASTDPGKVPRNWGFYIGDDVKRRRYC
PF3D7_0609800     51 SGSFTIAVTFHIFFILFILSFIKCASTDPGKVPRNWGFYVGDDVKRRRYC
PRCDC_0608500     51 TGSFTIAVTFHIFFILFILSFIKCASTDPGKVPRNWGFYVGDDVKRRRYC

                                             ****
PBANKA_010830    101 KICNVWKPDRTHHCSACNRCVLNMDHHCPWINNCVGFYNRRFFMQLLFYG
PCHAS_010890     101 KICNVWKPDRTHHCSACNRCVLNMDHHCPWINNCVGFYNRRFFMQLLFYG
PY17X_0109900    101 KICNVWKPDRTHHCSACNRCVLNMDHHCPWINNCVGFYNRRFFIQLLFYG
PKNH_1140400      96 KICNVWKPDRTHHCSACNRCVLNMDHHCPWINNCVGFFNRRFFIQLLFYG
PVX_113640       101 KICNVWKPDRTHHCSACNRCVLNMDHHCPWINNCVGFFNRRFFIQLLFYG
PF3D7_0609800    101 KICNVWKPDRTHHCSACNRCVLNMDHHCPWINNCVGFFNRRFFIQLLFYG
PRCDC_0608500    101 KICNVWKPDRTHHCSACNRCVLNMDHHCPWINNCVGFFNRRFFIQLLFYG

PBANKA_010830    151 LICLFMVATQTFHYIFIDNINAYMDKGFQENSSFVALEYTYASIVLFLTF
PCHAS_010890     151 LICLFMVATQTFHYIFIDNINAYMDTGFQENNSFVALEYTYASIVLFLTF
PY17X_0109900    151 LICLFMVATQTFHYIFIDNINAYMDTGFQENSSFVALEYTYASIVLFLTF
PKNH_1140400     146 LICLFIVAVQTFHYIFIDNANAYIEDGFHDKSSFVALEYTYASIVLFLTF
PVX_113640       151 LICLFMVALQTFYYIFIDNANAYIEDGFHDKSSFVALEYTYASIVLFLTF
PF3D7_0609800    151 LVCLFIIAVQTFHYIFIDNINAYFDDGFQEKSSFVALEYTYASIVLFLTF
PRCDC_0608500    151 LVCLFIIAVQTFHYIFIDNINAYFDDGFQEKSSFVALEYTYASIVLFLTF

PBANKA_010830    201 VLIFALVPFTKFHLKLISKNSTTIENMDIYHQDYNIYNVGCEDNAKQVFG
PCHAS_010890     201 VLIFALVPFTKFHLKLISKNSTTIENMDIYHQDYNIYNVGCEDNAKQVFG
PY17X_0109900    201 VLIFALVPFTKFHLKLISKNSTTIENMDIYHQDYNIYNVGCEDNAKQVFG
PKNH_1140400     196 VLIFALVPFTKFHLKLISKNSTTIENMDIYNQDYNMYNVGCEDNAKQVFG
PVX_113640       201 VLIFALVPFTKFHLKLISKNSTTIENMDIYNQDYNMYNVGCEDNAKQVFG
PF3D7_0609800    201 VLIFALVPFTKFHLKLISKNSTTIENMDMYSQEYNIYNVGCEDNAKQVFG
PRCDC_0608500    201 VLIFALVPFTKFHLKLISKNSTTIENMDMYSQEYNIYNVGCEDNAKQVFG

PBANKA_010830    251 NNILCWMCPCHCISNRPAGDGVRWRVSMSHGSNI
PCHAS_010890     251 NNILCWMCPCQCISNRPAGDGVRWRVSISQGTNI
PY17X_0109900    251 NNILCWMCPCHCISNRPAGDGVRWRVSISHGNNI
PKNH_1140400     246 NNILCWMCPFHCISNRPAGDGVRWRVSVAHENII
PVX_113640       251 NNILCWMCPFHCISNRPAGDGVRWRVSVAHENMI
PF3D7_0609800    251 NNILCWLCPFQCVSNRPAGDGVRWRVSVAHENPV
PRCDC_0608500    251 NNILCWLCPFQCVSNRPAGDGVRWRVSVAHENPV
```

Supplementary Figure S2. CLUSTAL W (1.83) multiple sequence alignment of DHHC2 from 7 *Plasmodium* species: PBANKA_010830 *Plasmodium berghei* ANKA; PCHAS_010890 *Plasmodium chabaudi chabaudi*; PY17X_0109900 *Plasmodium yoelii yoelii* 17X; PKNH_1140400 *Plasmodium knowlesi* strain H; PVX_113640 *Plasmodium vivax* Sal-1; PF3D7_0609800 *Plasmodium falciparum* 3D7; PRCDC_0608500 *Plasmodium reichenowi* CDC. Asterisk indicate position of the signature DHHC motif.

```
TGGT1_278850            1 MYHGPSPMAPGLLYHQPEGYAPAPTGPPLPQYNAPRGSGSAPPQAGMTSH
NCLIV_067160            1 MYHGPSPLGPTMLYHQPDGYAPAPLGP------------APMAAASSHQH
EfaB_MINUS_7900.g824    1 ------MYSPSPVFTAVHGRAPTPQGPP-----VALGPPGAPSNGNRAAV
PBANKA_010830           1 --------MTHKYMQISHGQP-----------------------------

TGGT1_278850           51 SALSPRGFPPSPTNRAPTRVPVSGAAG---TSRRSGNGCFLFAVVLLLSF
NCLIV_067160           39 SSTSPRGYPASPTAARGPAGLVPSAPVGATPPRKSGNACFLLAVVLLLSF
EfaB_MINUS_7900.g824   40 ASVSSAGVGNRMTGGPEGVGSSGPLGSLSHRRMGSGGGCFLFAVIFLMFF
PBANKA_010830          14 -------------------------------EKKRGGSIFIFIVFFILSF

TGGT1_278850           98 LYLGYVFILLAPLLWPIPSMLGSVLFVAFHCSFVLLLGAFLKAVCTDPGR
NCLIV_067160           89 LYLGYVFILLAPLLWPYPSMLGFVLFVAFHCCFVLLLGAFLKAVCTDPGR
EfaB_MINUS_7900.g824   90 LYFGYLTILLLPLLHESWSMFSVGLFVLFHVLFCLQLAAFFKAVCTDPGQ
PBANKA_010830          33 IYIGYTGIVLRSWFIPYRSGS-LTIAIVFHIFFCLFLLSFIKCASTDPGK

                                                                     ****
TGGT1_278850          148 VPANWGFYMGDENKRRRYCKVCNVWKPDRTHHCSACGRCVLNMDHHCPWI
NCLIV_067160          139 VPANWGFYMGDENKRRRYCKVCNVWKPDRTHHCSACGRCVLNMDHHCPWI
EfaB_MINUS_7900.g824  140 VPPHWGFYMGDESKRRRYCKVCNVWKPDRTHHCSACGRCVLNMDHHCPWI
PBANKA_010830          82 VPRNWGFYVGDDVKRRRYCKICNVWKPDRTHHCSACNRCVLNMDHHCPWI

TGGT1_278850          198 NNCVGFYNRKYFIQLLIYAIACLFFIFIHGFYFIFVESIR--STQTHPTA
NCLIV_067160          189 NNCVGFYNRKYFIQLLIYAIACLFFIFIHGFYFIFVESIR--STQPHS--
EfaB_MINUS_7900.g824  190 NNCVGFFNRRFFLQLLLYALACLTIVFIHTFHHIFVSSFYGLAERVGGPP
PBANKA_010830         132 NNCVGFYNRRFFMQLLFYGLICLFMVATQTFHYIFIDNIN----------

TGGT1_278850          246 LEHSVLSYEPDASAVAVLKYVYVCLMLFFSMVLIFALIPFSRFHLNLVLK
NCLIV_067160          235 PEIHALPYQTETSAAAVLKYVYVCLMLFFSMVLIFALIPFSRFHLNLVLK
EfaB_MINUS_7900.g824  240 PGRTLRPGEYSPSAFKVTEYIYSCLILFLSMLLIFALVPFTRFHLNLVLR
PBANKA_010830         172 --AYMDKGFQENSSFVALEYTYASIVLFLTFVLIFALVPFTKFHLKLISK

TGGT1_278850          296 NSTTIENMDVANRDRNRYDLGVSRNIEQVFGSNPCCWFVPVQFAANRPVG
NCLIV_067160          285 NSTTIENMDVANRDRNRYDLGVSRNIEQVFGSNPCCWFVPAQFAANRPVG
EfaB_MINUS_7900.g824  290 NSSTIENMDQSNFDRNRYDLGVGRNIEQVFGSNPCCWLLPTHAAGNRPVG
PBANKA_010830         220 NSTTIENMDIYHQDYNIYNVGCEDNAKQVFGNNILCWMCPCHCISNRPAG

TGGT1_278850          346 DGVRWNMHYMVADEHV-
NCLIV_067160          335 DGVRWNMHYMVADEQV-
EfaB_MINUS_7900.g824  340 DGVRWSLHYMVAQDTEA
PBANKA_010830         270 DGVRWRVSMSHGSNI--
```

Supplementary Figure S3. CLUSTAL W (1.83) multiple sequence alignment of *Plasmodium berghei* PBANKA_010830 and proteins from three related apicomplexan species: TGGT1_278850 *Toxoplasma gondii* GT1; NCLIV_067160 *Neospora caninum* Liverpool; EfaB_MINUS_7900.g824 *Eimeria falciformis* Bayer Haberkorn 1970. Note the high sequence conservation surrounding the central DHHC motif which is located within the loop region between transmembrane domains 2 and 3. Asterisk indicate position of the signature DHHC motif.

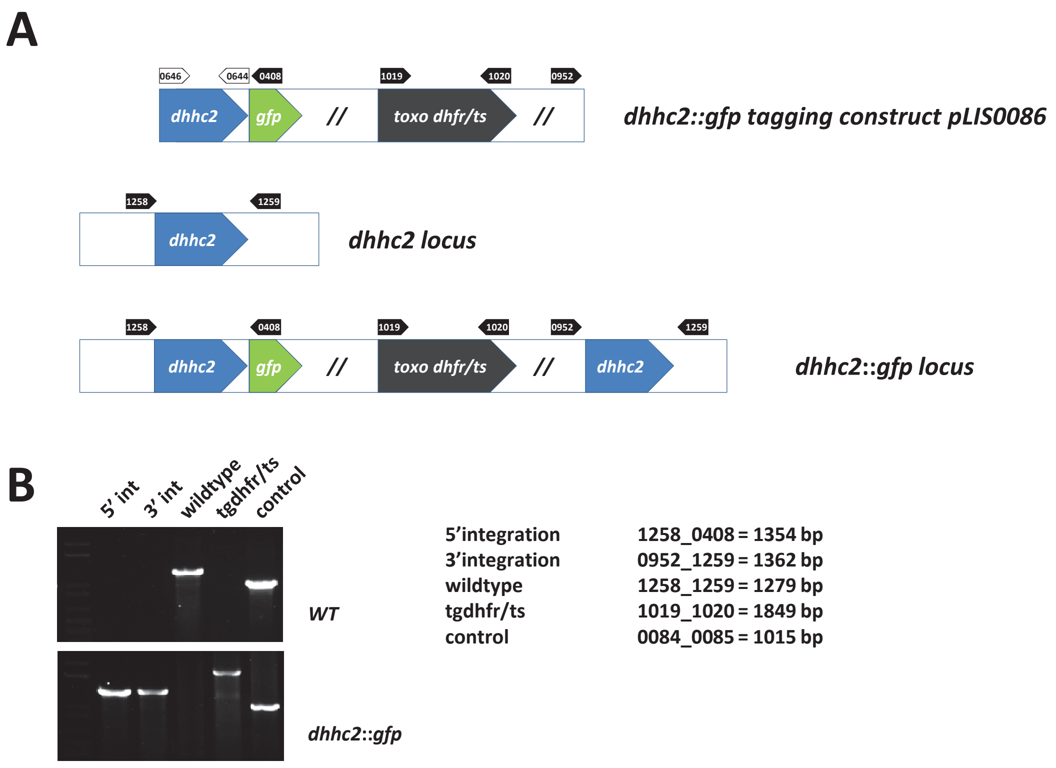

Supplementary Figure S4. Generation and genotyping of *dhhc2*::*gfp* parasite line. (A) *dhhc2* GFP tagging construct pLIS0086 was obtained by cloning the last 1167 bp of *dhhc2* ORF excluding the stop codon upstream and in frame with the *gfp* gene. This construct includes the *Toxoplasma gondii dhfr/ts* selectable marker cassette under the control of *P. berghei dhfr/ts* 5' and 3' UTRs. The construct was integrated into the *dhhc2 locus* of cl15cy1 by single homologous recombination, resulting in the fusion of *dhhc2* to *gfp* in *dhhc2*::*gfp* parasites. **(B)** Correct tagging of *dhhc2* was shown by PCR genotyping analyses. PCR analyses confirm 5' and 3' integration (int.) of pLIS0086, absence of WT *dhhc2* ORF and presence of *tgdhfr/ts* gene. Primer combinations and expected amplicon sizes in basepairs (bp) are shown on the right.

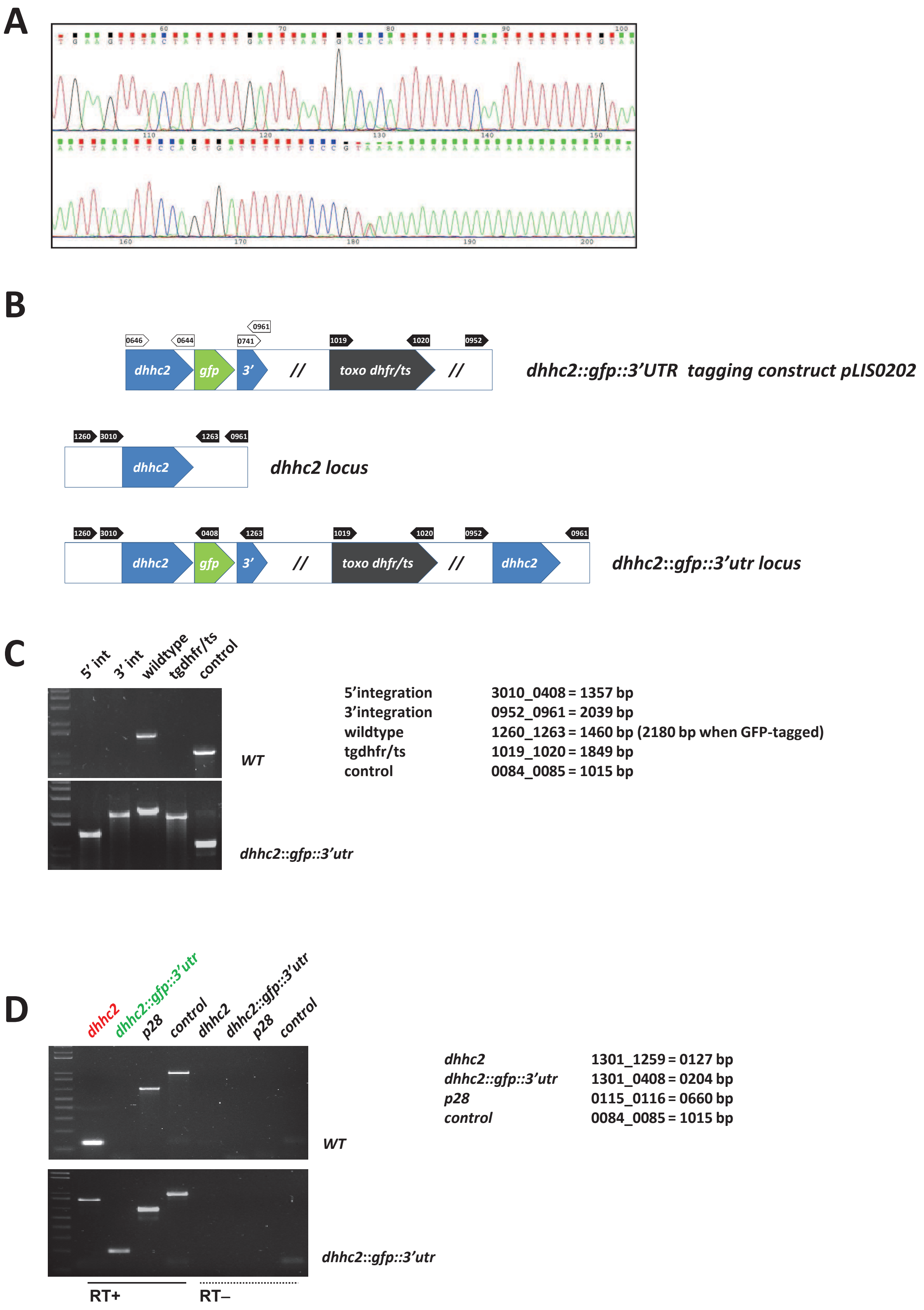
A
B
0646
0644
0961
0741
1019
1020
0952
dhhc2
gfp
3'
//
toxo dhfr/ts
//
dhhc2::gfp::3'UTR tagging construct pLIS0202
1260
3010
1263
0961
dhhc2
dhhc2 locus
1260
3010
0408
1263
1019
1020
0952
0961
dhhc2
gfp
3'
//
toxo dhfr/ts
//
dhhc2
dhhc2::gfp::3'utr locus
C
5' int
3' int
wildtype
tgdhfr/ts
control
WT
dhhc2::gfp::3'utr
5'integration 3010_0408 = 1357 bp
3'integration 0952_0961 = 2039 bp
wildtype 1260_1263 = 1460 bp (2180 bp when GFP-tagged)
tgdhfr/ts 1019_1020 = 1849 bp
control 0084_0085 = 1015 bp
D
dhhc2
dhhc2::gfp::3'utr
p28
control
dhhc2
dhhc2::gfp::3'utr
p28
control
WT
dhhc2::gfp::3'utr
RT+
RT–
dhhc2 1301_1259 = 0127 bp
dhhc2::gfp::3'utr 1301_0408 = 0204 bp
p28 0115_0116 = 0660 bp
control 0084_0085 = 1015 bp

Supplementary Figure S5. Generation and genotyping of *dhhc2*::*gfp::3'utr* parasite line. (A) The chromatogram shows the sequencing result of a 3' RACE product allowing determination of the length of the 3'UTR of *dhhc2*. **(B)** *dhhc2* GFP tagging construct pLIS0202 was obtained by cloning the last 1167 bp of *dhhc2* ORF excluding the stop codon upstream and in frame with the GFP gene and nucleotide positions 36 to 541 of the *dhhc2* 3' UTR downstream of *gfp*. This construct includes the *Toxoplasma gondii* dhfr/ts selectable marker cassette under the control of *P. berghei dhfr/ts 5'* and *3' utrs*. The construct was integrated into the *dhhc2* locus of *P. berghei* cl15cy1 by single homologous recombination, resulting in the fusion of *dhhc2* to *gfp* and maintaining the endogenous *dhhc2 3' utr* in *dhhc2::gfp-3'utr* parasites. **(C)** Correct tagging of *dhhc2* was shown by PCR genotyping analyses, confirming 5' and 3' integration (int.) of pLIS0202, absence of WT *dhhc2* ORF and presence of the *tgdhfr/ts* gene. **(D)** Absence of WT *dhhc2* and exclusive presence of *dhhc2::gfp* mRNA was confirmed in cloned *dhhc2::gfp-3'utr* mixed blood stages by RT-PCR.

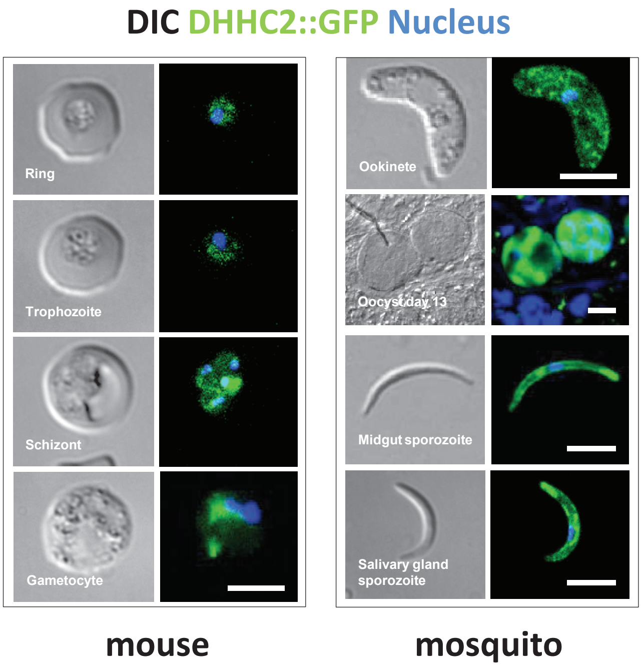

Supplementary Figure S6. Expression of DHHC2::GFP in *dhhc2::gfp::3'utr* parasites. Scale bars = 5 µm (blood stages, ookinetes and sporozoites) or 20 µm (oocysts).

Supplementary Figure S7. Transcript levels as reads per kilobase of exon model per million mapped reads (RPKM). Ring 4-hour ring stage; troph 16-hour trophozoite; schizont 22-hour schizont; G gametocytes; O 24-hour ookinete; Otto et al. 2014

PBANKA_140060 cytoadherence linked asexual protein, putative

4H R	209.626
16H T	479.428
22H S	2563.504
G	26.995
O	25.725

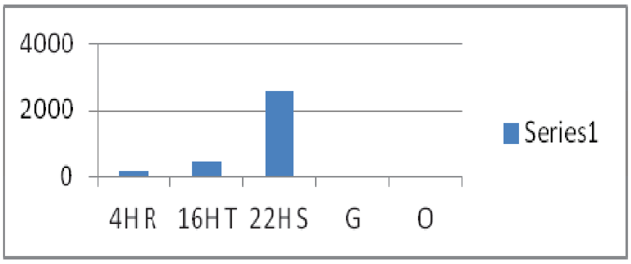

PBANKA_010830 palmitoyltransferase, putative (DHHC2)

4H R	24.294
16H T	3.636
22H S	255.355
G	283.007
O	29.628

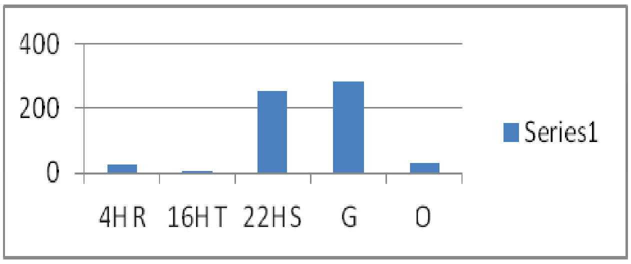

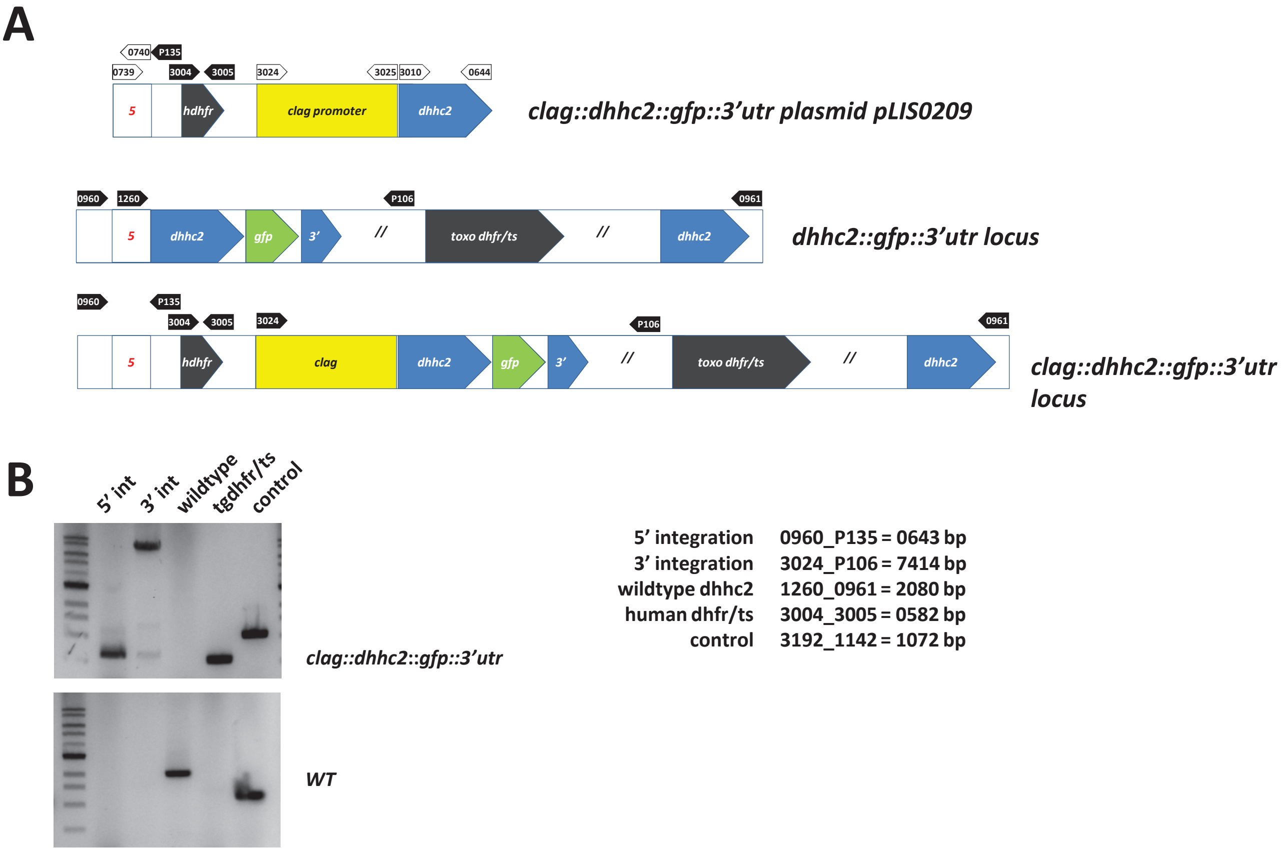

Supplementary Figure S8. Generation and genotyping of the *clag::dhhc2::gfp::3'utr* parasite line. (A) The *dhhc2* GFP promoter swap construct pLIS0209 was obtained by cloning the *clag* promoter in front of the *dhhc2* ORF. The human *dhfr* selectable marker cassette under the control of *P. berghei ef1a* 5' and *3' UTRs* is further flanked upstream by *dhhc2* upstream region and is thus integrated into the *dhhc2* locus of *dhhc2::gfp::3'utr* by double homologous recombination, resulting in the exchange of the endogenous *dhhc2* promoter for the *clag* promoter. **(B)** Correct promoter swap of *dhhc2* was shown by PCR genotyping analyses, confirming 5' and 3' integrations (int.) of pLIS0209, absence of WT *dhhc2* gene, and presence of the human *dhfr* gene.

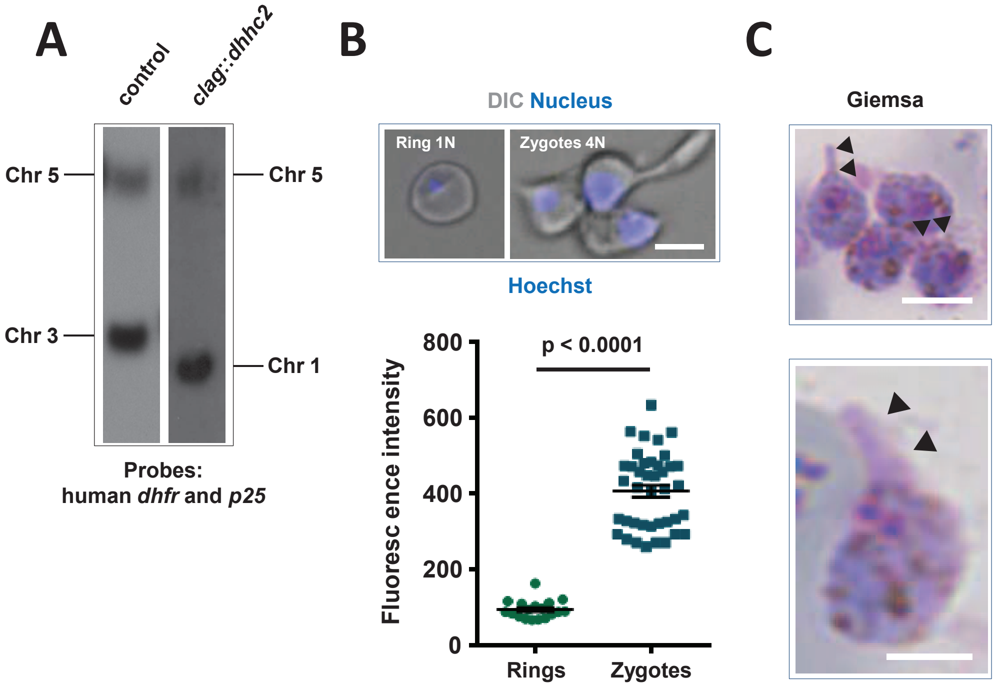

Supplementary Figure S9. ***clag::dhhc2*** **parasites (parental line: wildtype) are capable of normal fertilization but fail to form ookinetes, replicating the phenotype of** ***clag::dhhc2::gfp::3'utr*** **mutants. (A)** Construct pLIS0209 was integrated into the *dhhc2 locus* of parental wildtype reference line 676m1cl1 by double homologous recombination, resulting in the exchange of the endogenous *dhhc2* promoter for the *clag* promoter. Correct promoter swapping was shown by Southern analysis of separated chromosomes. Hybridisation of separated chromosomes was done with a mixture of two probes: one against the human *dhfr*, recognising integrated pLIS0209 into chromosome 1, and another against the gene *p25* on chromosome 5. As control, a transgenic parasite line containing the human *dhfr* in the *p230p locus* on chromosome 3 was used. **(B)** Live imaging of Hoechst-stained ring stage and zygotes of the *dhhc2* promoter swap line (scale bar=5 µm), and quantification of DNA content. Nuclear DNA content of asexual blood stage rings (haploid DNA content, 1N) and zygotes (tetraploid DNA content, 4N) was determined by Hoechst-fluorescence intensity measurements. The mean fluorescence intensity of ring-form nuclei (1N) is 94 (n=18) and of zygotes (4N) is 406 (n=40). Mean ± SEM values and p-value for Student's t-test are shown. **(C)** Giemsa staining of overnight ookinete cultures reveals 2-BMP-like morphological defects in mutant zygotes displaying thin protrusions (arrowheads). Scale bars=5 µm.

Table S1a - Primers used in the present work.

a) Primers used in life-cycle RT-PCRs, RT-PCRs of IP samples and 3'RACE

"c" or "Rev" at the end of primer names means they are antisense primers. All others are sense primers.
Nucleotide stretches in capital letter correspond to the complementary sequence to the respective gene.
n.a.: not applicable

Gene name	Gene ID	Primer name	Sequence	Description
dhhc2	PBANKA_010830	g0646	aaagaattcTCGATATTCATATTTATTG	*dhhc2* ORF
		g0644c	aaagcggccgcATATATTACTTCCATGTG	*dhhc2* ORF
***18S* rRNA**	n.a.	PbA18SFw	AAGCATTAAATAAAGCGAATACATCCTTAC	*18S* rRNA
		PbA18SRev	GGAGATTGGTTTTGACGTTTATGTG	*18S* rRNA
hsp70	PBANKA_071190	g0258	AAAAGCAAAGCCAAACTTACC	*hsp70* ORF
		g0259c	GGATGGGGTTGTTCTATTACC	*hsp70* ORF
p25	PBANKA_051500	g0385	CCGGAATTCATAAACAAATATACCTGG	*p25* 3' UTR
		g0476c	CGGGATCCTCATACGAATTTTATTG	*p25* 3' UTR
p28	PBANKA_051490	g0115	TTCGATATCATGAATTTTAAATACAG	*p28* ORF
		g0116c	tccgcggccgcGCATTACTATCACGTAAATAAC	*p28* ORF
dozi	PBANKA_121770	g0546	TAATTGTGTCGCTTCAAATG	*dozi* ORF
		g0548c	TAATTCTTTTATCATAGCAG	*dozi* ORF
cith	PBANKA_130130	g0549	GAAAAAAGCAAAGATGTATTATCTG	*cith* ORF
		g0550c	ATAGGCTGGGTATCTGTTAAATG	*cith* ORF
alba3	PBANKA_120440	g0003	aaacccggggaattcCAAGAAAGAGCTGAAAAC	*alba3* ORF
		g0004c	aaagcggccgctATTAGCAACAAAGTTTG	*alba3 ORF*
dhhc2	PBANKA_010830	g3315	TAATGCAAAACAGGTTTTTGG	*dhhc2 ORF*
		g1301	CATTGTATTTCAAATAGACC	*dhhc2 ORF*
		g3318	CAATACTTTTCAGTAATAAAATG	*dhhc2 3'utr*
		g3319	AAATCACTGGAATTTAATTTTAC	*dhhc2 3'utr*
**n/a**	oligo d(T)	g0357c	GCTGCAAGTGAACTATCTGTTTTTTTTTTTTTTTTT	*poly(A)*

Table S1b - Primers used in the present work.

b) Primers used in the generation of gene deletion and GFP-tagging constructs.
"c" at the end of primer names means they are antisense primers. All others are sense primers.
ORF: open reading frame; UTR: untranslated region.

Gene name	Gene ID	Construct name	Primer name	Sequence	Restriction sites
**Gene deletion constructs**					
dhhc2	PBANKA_010830	pLIS0065	g0739	aaaggtaccTTTATTATTTGAGTGTTG	*Asp718I*
			g0740c	aaaaagcttTTTATATTGATTTTGATTG	*HindIII*
			g0741	aaagaattcTTGAGTTTATAATATGTC	*EcoRI*
			g0742c	aaagcggccgcATATCCTAAAAACTATTG	*NotI*
**GFP-tagging constructs**					
dhhc2	PBANKA_010830	pLIS0086	g0646	aaagaattcTCGATATTCATATTTATTG	*EcoRI*
			g0644c	aaagcggccgcATATATTACTTCCATGTG	*NotI*
		pLIS0202	g0646	aaagaattcTCGATATTCATATTTATTG	*EcoRI*
			g0644c	aaagcggccgcATATATTACTTCCATGTG	*NotI*
			g0741	aaagaattcTTGAGTTTATAATATGTC	*EcoRI*
			g0742c	aaagcggccgcATATCCTAAAAACTATTG	*NotI*
**Promoter swap construct**					
**dhhc2**	**PBANKA_010830**	pLIS209	g3010	aaaactagtATGACACATAAATATATGCAGATAT	*SpeI*
			g0644c	aaagcggccgcATATATTACTTCCATGTG	*NotI*
**clag**	**PBANKA_140060**		g3024	aaacgatcgCTGATATTTATGAGTATTCC	*PvuI*
			g3025c	aaatctagaTATATTACGAATTGACACC	*XbaI*

Table S1c - Primers used in the present work.

c) Primers used in genotyping and RT-PCR of mutant parasite lines.
"c" at the end of primer names means they are antisense primers. All others are sense primers.
Nucleotide stretches in capital letter correspond to the complementary sequence to the respective gene.
n.a.: not aplicable; ORF: open reading frame; UTR: untranslated region.

Gene name/ mutant name	Gene ID	Primer name	Sequence
**Primers for genotyping**			
dhhc2::*gfp*	PBANKA_010830	g1258	ACACATAAATATATGCAG
		g1259c	ATGACATATTATAAACTC
dhhc2::*gfp*-3'UTR		3010	ATGACACATAAATATATGCAGATAT
		g0961c	GAGAATTAAGCCTATATATAC
		g1260	TGAGGAGAATATTATAAG
		g1263c	AATGCTATTGCATATTTG
dhhc2		g1258	ACACATAAATATATGCAG
		g1259c	ATGACATATTATAAACTC
		g1260	TGAGGAGAATATTATAAG
		g1263c	AATGCTATTGCATATTTG
**Primers for RT-PCR**			
dhhc2	PBANKA_010830	g1261	AATATATGCAGATATCAC
		g1262c	AAAAAAATATACTTGTGC
		g1301	CATTGTATTTCAAATAGACC
		g1259c	ATGACATATTATAAACTC
dhhc2::*gfp*		g1258	ACACATAAATATATGCAG
		g1301	CATTGTATTTCAAATAGACC
dhhc2::*gfp*-3'UTR		g1301	CATTGTATTTCAAATAGACC
		g1259c	ATGACATATTATAAACTC
**General primers**			
pbdhfr/*ts*	PBANKA_071930	g0952	GATTCATAAATAGTTGGACTTG
		P106	GTCCATACAACTATATCCGAAC
tgdhfr/*ts*	n.a.	g1019	ATGCATAAACCGGTGTGTC
		g1020c	AGCTTCTGTATTTCCGC
RNA polymerase II subunit RPB1	PBANKA_080700	g0084	aaagaattcTGATGGTTTACAATCACC
		g0085c	aaagcggccgctTTCTTCCTGCATCTCCTC
p28	PBANKA_051490	g0115	TTCGATATCATGAATTTTAAATACAG
		g0116c	tccgcggccgcGCATTACTATCACGTAAATAAC
gfp	n.a.	g0408c	GTATGTTGCATCACCTTC
ef1a	PBANKA_113330	P135	GTAAACTTAAGCATAAAGAGCTCG
human dhfr	n.a.	g3004	AAAAGATCTATGGTTGGTTCGCTAAACTG
human dhfr	n.a.	g3005	AAACAATTGTTAATCATTCTTCTCATATAC

Supplementary Table S2. Parasite transfection experiments overview

Gene name/ mutant name	Gene ID	DNA construct name	Restriction enzymes[1]	Experiment #/ mutant clone ID[2]	Parental line[3]
**Unsussessful gene deletion attempts**					
dhhc2	PBANKA_010830	pLIS0065	*Asp*718I and *Not*I	2095	820cl1m1cl1
				2362, 2363	676m1cl1
**GFP-tagged mutants**					
dhhc2::gfp	PBANKA_010830	pLIS0086	*Fsp*AI	2185cl1m1	cl15cy1
dhhc2::gfp-3'utr	PBANKA_010830	pLIS0202	*BsmI*	202cl1m1	cl15cy1
**Promoter swap**					
clag::dhhc2::gfp::3'utr	PBANKA_010830	pLIS209	*KpnI, BglI*	209cl2	202cl1m1
clag::dhhc2	PBANKA_010830	pLIS209	*KpnI, BamHI*	2593	676cl1m1

[1] Restriction enzymes used for plasmid linearisation before transfection

[2] Experiment number for independent transfection experiments:
the unsuccessful attempts and the experiment number/ID of the mutants clones

[3] Parental *P. berghei* ANKA line in which the transfection experiment was performed
